# Genome-wide meta-analysis identifies novel loci associated with parathyroid hormone level

**DOI:** 10.1186/s10020-018-0018-5

**Published:** 2018-04-11

**Authors:** Antonela Matana, Dubravka Brdar, Vesela Torlak, Thibaud Boutin, Marijana Popović, Ivana Gunjača, Ivana Kolčić, Vesna Boraska Perica, Ante Punda, Ozren Polašek, Maja Barbalić, Caroline Hayward, Tatijana Zemunik

**Affiliations:** 1 Department of Medical Biology, University of Split, School of Medicine, Šoltanska 2, Split, Croatia; 2 Department of Nuclear Medicine, University Hospital Split, Spinciceva 1, Split, Croatia; 3 MRC Human Genetics Unit, University of Edinburgh, Western General Hospital, Crewe Road, Edinburgh, UK; 4 Department of Public Health, University of Split, School of Medicine Split, Šoltanska 2, Split, Croatia

**Keywords:** Parathyroid hormone, Genome-wide association analysis, Meta-analysis

## Abstract

**Background:**

Parathyroid hormone (PTH) is one of the principal regulators of calcium homeostasis. Although serum PTH level is mostly accounted by genetic factors, genetic background underlying PTH level is insufficiently known*.* Therefore, the aim of this study was to identify novel genetic variants associated with PTH levels.

**Methods:**

We performed GWAS meta-analysis within two genetically isolated Croatian populations followed by replication analysis in a Croatian mainland population and we also combined results across all three analyzed populations. The analyses included 2596 individuals. A total of 7,411,206 variants, imputed using the 1000 Genomes reference panel, were analysed for the association. In addition, a sex-specific GWAS meta-analyses were performed.

**Results:**

Polymorphisms with the lowest *P*-values were located on chromosome 4 approximately 84 kb of the 5′ of *RASGEF1B* gene. The most significant SNP was rs11099476 (*P* = 1.15 × 10^−8^). Sex-specific analysis identified genome-wide significant association of the variant rs77178854, located within *DPP10* gene in females only (*P* = 2.21 × 10^− 9^). There were no genome-wide significant findings in the meta-analysis of males.

**Conclusions:**

We identified two biologically plausible novel loci associated with PTH levels, providing us with further insights into the genetics of this complex trait.

**Electronic supplementary material:**

The online version of this article (10.1186/s10020-018-0018-5) contains supplementary material, which is available to authorized users.

## Background

Parathyroid hormone (PTH) plays a critical role in the regulation of bone mineral metabolism and calcium homeostasis (DeLuca, 1986). PTH regulates serum calcium levels by stimulating osteoclast activity within bone in order to release calcium. Circulating PTH enhances the reabsorption of calcium in distal nephrons and induces the synthesis of the vitamin D active metabolite 1,25-dihydroxyvitamin D (1,25(OH)_2_D_3_) within the kidney (Kumar & Thompson, 2011; Kumar et al., 1991; Khundmiri et al., 2016). The 1,25(OH)_2_D_3_ stimulates intestinal calcium absorption and moreover, has a synergistic effect with PTH in bone resorption by stimulating proliferation of osteoclasts (Kumar & Thompson, 2011; Kumar et al., 1991; Khundmiri et al., 2016).

Variations in PTH synthesis and secretion are regulated by serum levels of calcium and phosphate, as well as by 1,25(OH)_2_D_3_ (Kumar & Thompson, 2011; Gago et al., 2005). Decreases in serum levels of calcium and increases in serum levels of phosphate stimulate the secretion of PTH, while 1,25(OH)_2_D_3_ decreases PTH secretion (Silver & Levi, 2005). Regulation of PTH secretion in response to variations in serum calcium is mediated by the calcium-sensing receptors on the membrane of parathyroid cells (Kumar & Thompson, 2011; Brent et al., 1988). 1,25(OH)_2_D_3_ associates with the vitamin D receptor and thus represses the transcription of PTH. The secretion of PTH is also indirectly altered by 1,25(OH)_2_D_3_ and its regulation of calcium-sensing receptor expression (Kumar & Thompson, 2011). Serum phosphate regulates PTH mRNA and serum PTH levels independently of changes in either serum calcium or 1,25(OH)_2_D_3_ levels (Kilav et al., 1995).

The most common pathological condition of excessive secretion of parathyroid hormone is hyperparathyroidism. Primary hyperparathyroidism is due to hypersecretion of the parathyroid gland, while secondary hyperparathyroidism can result from conditions that lead to hypocalcemia, especially observed in patients with chronic kidney disease (Fraser, 2009). Hypoparathyroidism, parathyroid hormone deficiency, is an uncommon condition that occurs mostly due to surgical removal of the parathyroid gland (Abate & Clarke, 2017).

Both environmental and genetic factors influence serum PTH levels. It is estimated that 60% of the variation in PTH concentrations is genetically determined. (Hunter et al., 2001). However, the genetic background underlying PTH level is not yet well understood.

Only one high-density genome-wide association study (GWAS) of PTH concentration has been reported to date (Robinson-Cohen et al., 2017). Robinson-Cohen et al. identified five significantly associated loci, including the strongest associated SNP rs6127099 located upstream of *CYP24A1*, a gene that encodes the primary catabolic enzyme for 1.25 (OH)_2_D (Robinson-Cohen et al., 2017). The other significantly associated loci were intronic variant rs4074995 within *RGS14* (regulator of G-protein signaling 14), rs219779 adjacent to *CLDN14* (Claudin 14), rs4443100 located near *RTDR1* (RSPH14, radial spoke head 14 homolog) and rs73186030 located near *CASR* (calcium-sensing receptor) gene (Robinson-Cohen et al., 2017). However, only three of these five loci (rs6127099, rs4074995 and rs219779) were replicated within an independent sample. Altogether, the five reported loci explained only 4.2% of the variance in circulating PTH, suggesting that additional genetic variants remain undiscovered.

The aim of our study is identification of novel loci associated with the parathyroid function, by performing a GWAS meta-analysis of plasma PTH levels within two genetically isolated Croatian populations (Korcula and Vis) following by replication analysis in the urban population of Split. To maximize the power of the study, we additionally performed meta-analysis for PTH plasma levels in all three Croatian populations. We also conducted gender-specific GWAS meta-analyses.

## Methods

### Study cohorts

This study was performed on samples from three Croatian populations: from the Dalmatian islands of Korcula and Vis and the mainland city of Split, within the large-scale project of “10,001 Dalmatians” (Rudan et al., 2009). A detailed description of the cohorts is provided in Table 1. The Korcula population is genetically isolated from Croatian Mainland, while Vis population is genetically isolated from Croatian Mainland and surrounding islands (Vitart et al., 2008). For all study populations, we excluded participants who underwent parathyroid surgery, as well as individuals who had PTH level < 5 pg/ml, which is near the minimum PTH assay detection limit (4.3 pg/ml). After these exclusions, the number of individuals available with PTH level and genotype data was 806 in Korcula, 831 in Vis and 959 in Split. In all three cohorts there were no participants who reported serious renal disease that could affect PTH concentration. The study was approved by the Research Ethics Committees in Croatia and Scotland and all participants provided informed consent. All analyses were in accordance with the relevant guidelines and regulations.

**Table 1:** Characteristics of study participants

Variables	Korcula	Vis	Split
N with PTH and GWAS data	863	834	960
N underwent parathyroid surgery	1	0	1
N with PTH level < 5 pg/ml	56	3	3
Sample size used in the analyses	806	831	959
Women, N (%)	524 (65%)	486 (58%)	586 (61%)
Median age, (q_L_,q_U_)	57 (47, 67)	57 (45,69)	52 (40, 61)
Median PTH, pg/ml (q_L_,q_U_)	19.9 (13.7, 29.1)	25.9 (18.4, 32.1)	21.6 (17.2, 26.5)

N: number of individuals; q_L:_ lower quartile, q_u:_ upper quartile

### Genotyping and imputation

Additional file 1: Table S1 shows cohort-summary information on genotyping, imputation and quality control procedures. The final numbers of single nucleotide polymorphisms (SNPs) included in analyses were 9,182,797 for the Korcula sample, 8,865,173 for the Vis sample and 8,777,560 for the Split sample. The number of overlapping SNPs present in all three cohorts was 7,411,206.

### Measurement of PTH

Plasma PTH levels were determined by radio-immunoassay method (RIA) in the Laboratory of Biochemistry, Department of Nuclear Medicine, University Hospital Split. RIA ran on the Scintillation counter liquid samples, Capintec, and 125I served as a marker. The concentrations of PTH in the plasma were determined using commercial kits (DIAsource hPTH -120 min-IRMA Kit, DIAsource ImmunoAssays S.A, Belgium). The reference range of plasma PTH levels is 12.26–35.50 pg/ml.

### Statistical analyses

We performed genome-wide association analysis within each data set and then conducted a meta-analysis of two genetically isolated cohorts (Korcula and Vis) followed by replication analysis in the cohort of the mainland city of Split. To maximize the study power, we also performed a further meta-analysis of all three cohorts.

### Genome-wide association analyses

Association analysis for the Split sample was carried out using a combination of R-package GenABEL and SNPTEST software, while for the Korcula and Vis samples analyses were conducted using R-packages GenABEL and VariABEL (Aulchenko et al., 2007; Marchini et al., 2007; Struchalin et al., 2012).

PTH levels were adjusted for age and sex using linear regression analysis and the calculated residuals were inverse-Gaussian transformed to achieve a normal distribution. GWAS was performed on transformed residuals using linear mixed model which accounts for population structure and relatedness. Association statistics for each SNP, including effect size estimates (β-estimates), standard errors and *p*-values were calculated under an additive genetic model.

Prior to performing the meta-analysis we calculated genomic inflation factors (lambdas) in individual data sets. No adjustments were necessary (*λ*_*Korcula*_ = 1.026, *λ*_*Vis*_ = 1.001, *λ*_*Split*_ = 0.99).

### Meta-analysis

Meta-analysis was carried out using the R-package MetABEL (R: A Language and Environment for Statistical Computing, 2018). Meta-analysis was conducted using the inverse-variance fixed-effects method on overlapping SNPs based on the β-estimates and standard errors from each study. Meta-analyses showed no significant evidence for inflated statistics (both *λ*_*Korcula* − *Vis*_ and *λ*_*Korcula* − *Vis* − *Split*_ were 1.01), thus no genomic correction was applied. To visualize results of the meta-analysis, Manhattan and quantile-quantile (QQ) plots were created using R-package qqman (Turner, 2014). Regional association plots for loci of interest (±400 kb) were produced using Locus Zoom based on hg19 genome build and 1000 genomes EUR population as the linkage disequilibrium (LD) population (Pruim et al., 2010). Forest plots for the most associated SNP were created using R-package MetABEL. To confirm the genotyping quality for the most associated SNPs in the regions, cluster plots were visually inspected using the Illumina GenomeStudio software package. If the SNP of interest was not directly genotyped, but imputed, then cluster plots were examined for directly genotyped SNPs in high LD with the SNP of interest (*r*^2^ > 0.8), located on the same chromosome and less than 400 kb apart. A genome-wide significance of association was defined as *p* − *value* ≤ 5 × 10^−8^. Power calculations were performed using Quanto version 1.2.4 for quantitative traits (WJ MJ, 2006).

### Sex-specific analyses

In order to identify sex-specific effects we performed GWAS analyzing males and females separately in each cohort. We used the same procedures as in the primary analyses with the exception of the gender covariate. Association results were meta-analyzed using the inverse-variance fixed-effects method. The total sample sizes were 1596 in women and 1000 in men.

## Results

### Meta-analyses

In each population, a separate genome-wide association study of PTH levels was conducted. We meta-analyzed two genetically isolated cohorts, Korcula and Vis (Additional file 1: Figure S1), and then replicated results in the Split population. A total of 1637 individuals were included in the meta-analysis and 959 in the replication analysis (Table 1). The most associated SNP was rs4616742 (reference allele C, *β* = 0.18, *SE* = 0.04, *P* = 4.42 × 10^−7^). The SNP is located near protein coding gene *RASGEF1B* (RASGEF Domain Family Member 1B). All top SNPs, located on the chromosome 4 near *RASGEF1B* gene with *P* < 10^−6^ from meta-analysis of ‘genetically isolated populations’ reached *P* < 0.05 in the Split population.

**Fig. 1:**
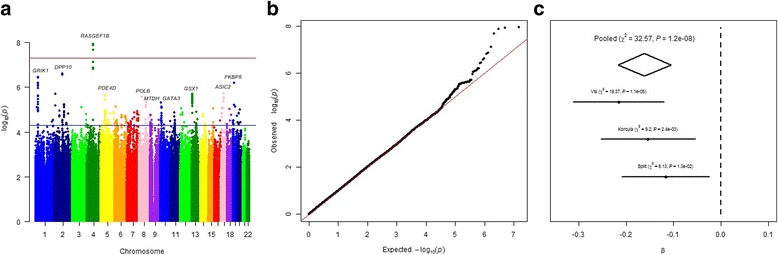
**a** Manhattan plot of SNPs for PTH levels in the meta-analysis of three cohorts. The *y* axis shows the −log_10_ *P* values of 7,411,206 SNPs, and the *x* axis shows their chromosomal positions. The blue line indicates the threshold for suggestive hits (*P* = 5 × 10^−5^), and the red line represents the threshold for genome-wide significance (*P* = 5 × 10^−8^). **b** Quantile-quantile plot in the meta-analysis of three cohorts. **c** Forest plot of rs11099476 effect estimates in individual populations and the combined meta-analysis

**Fig. 2:**
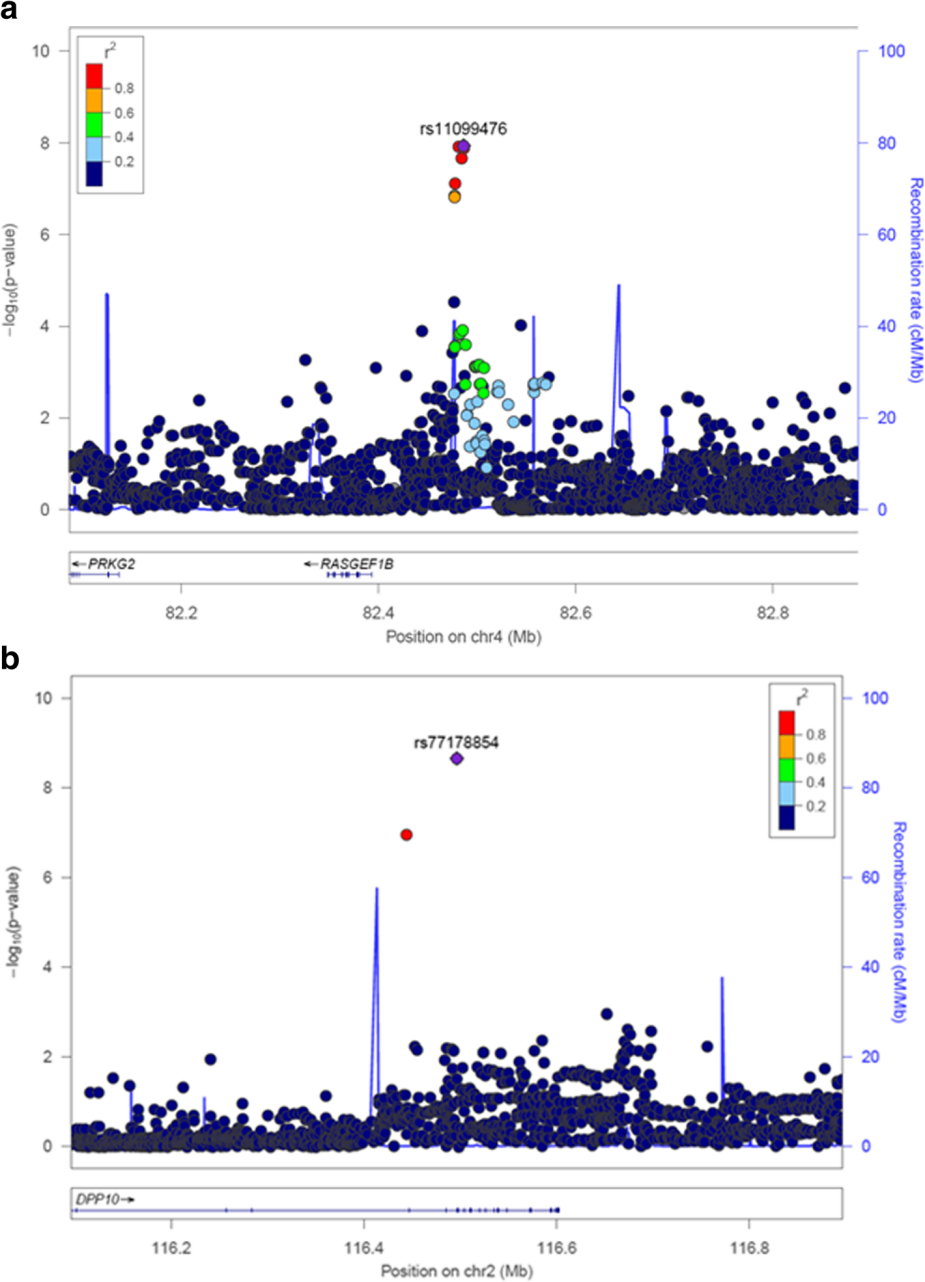
**a** Regional association plot for the chromosome 4 locus rs11099476 in the meta-analysis of three cohorts. **b** Regional association plot for the chromosome 2 locus rs77178854 in the sex-stratified meta-analysis of three cohorts among females. SNPs are plotted by position against association with PTH (−log_10_ *P* values). The purple diamond highlights the most significant, whereas the colors of other variant represent LD with most significant SNP

To maximize the study power, we performed a meta-analysis of all three cohorts. In total, 2596 individuals were included in the meta-analysis (Table 1). The results are shown in Fig. 1a. As seen from the quantile-quantile plot there was no early deviation from expected *P* values (Fig. 1b). Four SNPs, representing one locus, reached genome-wide significance. As in the meta-analysis of two genetically isolated cohorts, SNPs with the lowest *P*-values were located on chromosome 4 near *RASGEF1B*. The most associated SNP was rs11099476 (*P* = 1.15 × 10^−8^), which explained 1.14% of the variance in PTH. We found the T allele of the rs11099476 to be associated with higher PTH level (*β* = 0.16, *SE* = 0.03). Effect sizes were in the same direction across all three cohorts (Fig. 1c). The regional association plot for rs11099476 is given in Fig. 2a. The identified SNP, rs11099476, is in high LD with the top SNP from meta-analysis of ‘genetically isolated populations’, rs4616742 (r^2^ = 0.9). These results indicated that associated locus is becoming more significant as the sample size increases and confirmed the consistency of our top finding.

Analysis also revealed several suggestive loci (*P* < 5 × 10^−6^), including rs77178854 in the *DPP10* gene (*P* = 2.46 × 10^−7^), rs481121 near the *GRIK3* gene (*P* = 3.58 × 10^−7^), rs76615278 in the *FKBP8* gene (*P* = 6.34 × 10^−7^), rs1875872 near the *ASIC2* gene (*P* = 1.94 × 10^−6^), rs9512841 near the *GSX1* gene (*P* = 2.01 × 10^−6^), rs191686630 in the *PDE4D* gene, rs3136797 in the *POLB* gene (*P* = 2.68 × 10^−6^), rs499177 near the *MTDH* gene and rs58726672 near the *GATA3* gene (*P* = 4.77 × 10^−6^) (Table 2).

**Table 2:** Associations of top single nucleotide polymorphisms(*P* < 5 × 10^−6^) with PTH concentrations

SNP	Chr	Position	Nearest Gene	Effect Allele	Other Allele	EAF Korcula	EAF Vis	EAF Split	GnomAD EAF	β	SE	*P* value
rs11099476	4	82,486,056	*RASGEF1B*	T	A	0.57	0.55	0.54	0.59	0.16	0.03	1.15×10^−8^
rs77178854	2	116,496,539	*DPP10*	C	G	0.97	0.98	0.99	0.98	0.58	0.11	2.46×10^−7^
rs481121	1	37,203,485	*GRIK1*	A	G	0.56	0.56	0.58	0.49	0.14	0.03	3.58×10^−7^
rs76615278	19	18,654,588	*FKBP8*	G	A	0.84	0.83	0.82	*	0.20	0.04	6.34×10^−7^
rs1875872	17	31,795,716	*ASIC2*	A	G	0.62	0.65	0.65	0.65	0.14	0.03	1.94×10^−6^
rs9512841	13	28,309,646	*GSX1*	G	A	0.51	0.52	0.53	0.58	0.13	0.03	2.01×10^− 6^
rs191686630	5	58,477,398	*PDE4D*	A	T	0.11	0.16	0.21	*	0.19	0.04	2.36×10^−6^
rs3136797	8	42,226,805	*POLB*	C	G	0.98	0.99	0.98	0.99	0.57	0.12	2.68×10^−6^
rs499177	8	98,472,201	*MTDH*	T	C	0.46	0.57	0.45	0.44	0.13	0.03	4.66×10^−6^
rs58726672	10	8,407,822	*GATA3*	C	T	0.98	0.98	0.98	0.98	0.57	0.13	4.77×10^−6^

Top SNPs were defined as the SNP with lowest P value within a 500 kb window

Chr: chromosome; EAF: effect allele frequency; GnomAD EAF: effect allele frequency from Genome Aggregation Database; β: effect size; SE: standard error

*variants without frequency information in Genome Aggregation Database

### Sex-specific analyses

**Fig. 3:**
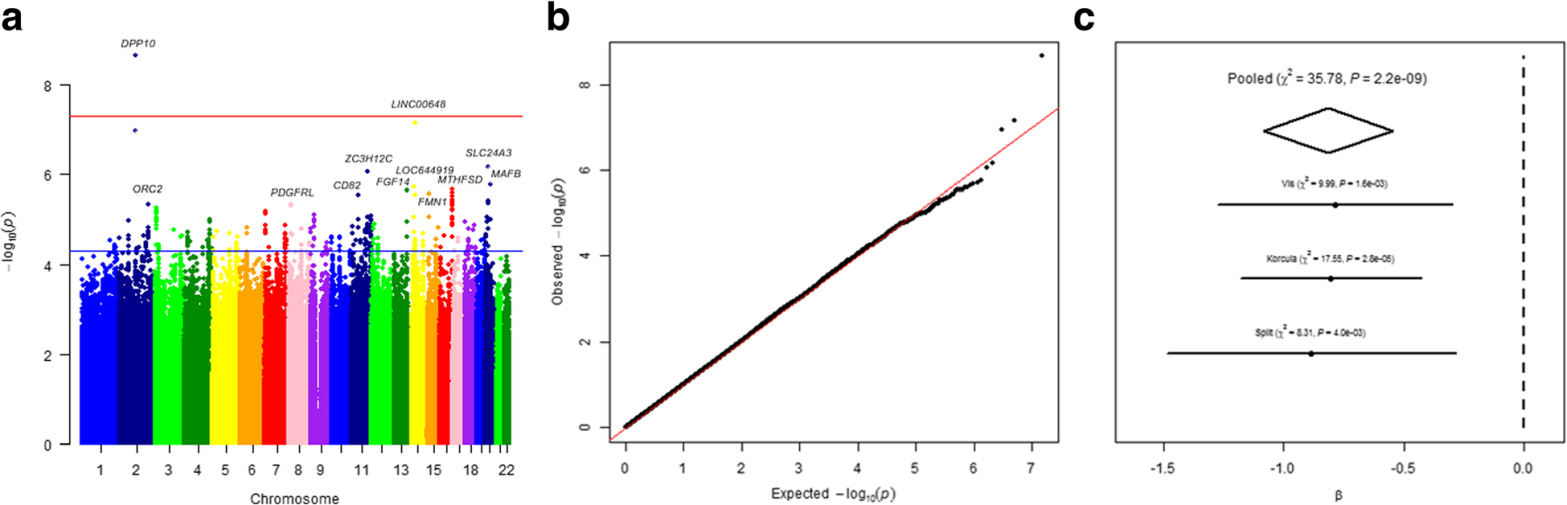
**a** Manhattan plot of SNPs for PTH levels in the sex-stratified meta-analysis of three cohorts among females. The *y* axis shows the −log_10_ *P* values of 7,411,206 SNPs, and the *x* axis shows their chromosomal positions. The blue line indicates threshold for suggestive hits (*P* = 5 × 10^−5^), and red line represents the threshold for genome-wide significance (*P* = 5 × 10^−8^). **b** Quantile-quantile plot in the sex-stratified meta-analysis of three cohorts among females. **c** Forest plot of rs77178854 effect estimates in individual populations and the combined meta-analysis among females

We searched for gender-specific loci by performing sex-specific GWAS meta-analysis, analyzing females and males separately in each cohort. The results for females are shown in Fig. 3. The top hit detected in the meta-analysis of all three cohorts, rs77178854, located within *DPP10* gene, reached genome-wide significance (reference allele C, *β* = 0.82, *SE* = 0.14, *P* = 2.21 × 10^−9^) in females (Table 3). Effect sizes were in the same direction across all three cohorts (Fig. 3c). Regional association plot of the identified SNP is shown in Fig. 2b. No single locus reaching genome wide significance was identified in males (Additional file 1: Table S2).

**Table 3:** Associations of top single nucleotide polymorphisms(*P* < 5 × 10^−6^) with PTH concentrations among females

SNP	Chr	Position	Nearest Gene	Effect Allele	Other Allele	EAF Korcula	EAF Vis	EAF Split	GnomAD EAF	β	SE	*P* value
rs77178854	2	116,496,539	*DPP10*	C	G	0.98	0.97	0.99	0.98	0.82	0.14	2.21×10^−9^
rs1890709	14	49,101,833	*LINC00648*	A	G	0.38	0.30	0.33	0.31	0.20	0.04	7.12×10^−8^
rs16981087	20	19,739,954	*SLC24A3*	G	C	0.80	0.78	0.77	0.81	0.22	0.04	6.99×10^−7^
rs661171	11	110,016,519	*ZC3H12C*	G	T	0.74	0.70	0.71	0.70	0.20	0.04	8.94×10^−7^
rs74629672	20	39,105,870	*MAFB*	T	A	0.95	0.94	0.94	0.96	0.43	0.09	1.68×10^−6^
rs1349573	14	41,403,160	*LOC644919*	G	A	0.05	0.05	0.06	0.03	0.45	0.10	1.94×10^−6^
rs3866634	16	86,567,929	*MTHFSD*	G	A	0.93	0.92	0.93	0.91	0.32	0.07	2.14×10^−6^
rs7997888	13	102,759,325	*FGF14*	A	G	0.04	0.04	0.04	0.15	0.49	0.10	2.19×10^−6^
rs5024438	15	33,077,401	*FMN1*	G	A	0.72	0.70	0.79	*	0.23	0.05	2.76×10^−6^
rs77796218	11	44,580,581	*CD82*	C	T	0.97	0.96	0.97	0.98	0.49	0.10	2.84×10^−6^
rs13406545	2	201,792,123	*ORC2*	T	A	0.15	0.19	0.18	0.23	0.21	0.05	4.54×10^−6^
rs2588129	8	17,462,468	*PDGFRL*	A	G	0.04	0.02	0.02	0.11	0.54	0.12	4.57×10^−6^

Top SNPs were defined as the SNP with lowest P value within a 500 kb window

Chr: chromosome; EAF: effect allele frequency; GnomAD EAF: effect allele frequency from Genome Aggregation Database; β: effect size; SE: standard error

*variants without frequency information in Genome Aggregation Database

## Discussion

In this GWAS meta-analysis of three Croatian populations we identified a novel genome-wide significant locus associated with plasma PTH level near gene *RASGEF1B* on chromosome 4. We also identified a sex-specific significant association in females in the *DPP10* gene.

The significance of the identified polymorphism rs11099476 was most influenced by the Vis population, which is isolated from the Croatian Mainland and surrounding islands, then by the Korcula population which is isolated from the Croatian Mainland and the least contributed by the mainland city of Split population (Fig. 2c). However, although the locus significance was most affected by the isolated populations, significance has been amplified in the meta-analysis of all three cohorts compared to meta-analysis of ‘genetically isolated populations’.

The identified common variant rs11099476 accounts for 1.14% of population variance in plasma PTH. *RASGEF1B* is the guanine nucleotide exchange factor with specificity for Rap2A, a member of Rap subfamily of Ras-like G proteins (Yaman et al., 2009). Rap2 subfamily contains Rap2A and Rap2B, which share about 90% sequence homology (Paganini et al., 2006). Rap2A protein binds GDP to GTP and exhibits a low intrinsic GTPase activity in the presence of Mg^2+^ (Lerosey et al., 1991), while Rap2B increases intracellular calcium level and phosphorylation level of extracellular signal-related kinase (ERK) 1/2 (Di et al., 2015). Variations near *RASGEF1B* gene have been associated with height (He et al., 2015; Allen et al., 2010). Height is positively correlated with calcium absorption efficiency which is important determinant of calcium balance (Abrams et al., 2005). Some evidence of association was also found for variations in this gene and bone density, hip, and cystatin C in serum (Kiel et al., 2007; Kottgen et al., 2010). PTH is a significant negative predictor of bone mineral density at the hip (Sneve et al., 2008). Cystatin C in serum is a biomarker of kidney function, and chronic kidney disease (Kottgen et al., 2010). Disturbed kidney function can influence PTH stimulated calcium reabsorption and synthesis of 1,25(OH)_2_D_3_ (Kumar & Thompson, 2011; Kumar et al., 1991; Khundmiri et al., 2016). However, to understand the mechanism underlying the observed association further functional studies of *RASGEF1B* will be needed.

Although no signals other than rs11099476 reached genome-wide significance, several candidate loci showed suggestive evidence of association. Particularly interesting is the variant near *GATA3* gene since mutations in this gene are the cause of hypoparathyroidism with sensorineural deafness and renal dysplasia (Van Esch et al., 2000). Of note, in a previous large GWA meta-analysis, variant near *GATA3* gene was found to be associated with serum calcium (O'Seaghdha et al., 2013).

Given the reported differences in PTH level between males and females, we performed sex-specific analyses (Wei et al., 2015; Serdar et al., 2017). Our study supports the sex-specificity underlying PTH level. Sex-stratified analysis in women identified a novel locus associated with PTH. The identified locus is the intron variant rs77178854, located within *DPP10* gene. *DPP10* encodes a membrane protein that is a member of the serine proteases family, which binds specific voltage-gated potassium channels and alters their expression and biophysical properties**.** It is highly expressed in brain, pancreas, spinal cord and adrenal gland (Allen et al., 2003), and may serve as a prognostic marker in colorectal cancer (Park et al., 2013). It is interesting to note that Aigner et al. showed that high serum PTH concentrations were associated with distal colorectal cancer in women but not in men (Aigner et al., 2015). The existence of *DPP10* in endocrine cells indicate that the protein might also have an additional role in the regulation of hormone secretion (Bezerra et al., 2015), which also supports our finding. Further studies of *DPP10* will be needed to clarify this result.

The only previously published high-density GWAS for PTH levels did not identify *RASGEF1B* or *DPP10* at a genome-wide significant level, despite having a sample size of over 29,155 participants (22, 653 in discovery stage and 6502 in replication analysis) (Robinson-Cohen et al., 2017). The possible explanation could be an increased relative effect of these loci in our populations due to the reduced genetic and environmental heterogeneity found in two out of three cohorts (i.e., Korcula and Vis) (Rudan et al., 2008) compared to the urban populations used in the analysis of Robinson-Cohen et al. *(Robinson-Cohen et al.,* 2017*)*. Previously reported *CYP24A1*, *RGS14* and *CLDN14* variants associated with PTH level (Robinson-Cohen et al., 2017) had the same directions of effect in our study as originally reported but did not show significant associations, probably due to limited sample size of our study or specificity of isolated populations (Additional file 1: Table S3).

The greatest strengths of our study include a comprehensive set of genetic variants examined and ethnically homogeneous sample. We had sufficient data to confidently detect an association for the identified *RASGEF1B* locus, since our meta-analysis had 92% power to detect associated SNP with an effect size of 0.19 and minor allele frequency of 0.45 at the genome-wide level of significance. Furthermore, meta-analysis performed in females only had 86% power to detect *DPP10* locus with an effect size of 0.82 and minor allele frequency of 0.02 at the genome-wide level of significance. Meta-analysis performed in males only had 84% power to detect SNPs with an effect size of 0.35 and minor allele frequency of 0.3 at the genome-wide level of significance. The main limitation of our study is the modest sample size used in the analysis, reducing statistical power for detecting additional associations with smaller effect sizes or minor allele frequencies. Nevertheless, we have identified novel, previously unsuspected and biological plausible associations with PTH variation. Further replication analysis would be required to confirm our findings and to discover additional genetic variants underlying PTH levels in order to explain more of the variability in PTH variations.

## Conclusions

In summary, in a GWA meta-analysis of PTH levels we identified a novel significant locus rs11099476 located near a guanine nucleotide exchange factor *RASGEF1B*. The finding appears to be consistent based on analyses of meta-GWAS across all three analyzed cohorts and meta-GWAS across two isolated populations followed by replication analysis in the mainland cohort. Our work also includes the first gender-specific GWAS performed to date and revealed significant association for an intron variant rs77178854 located within the *DPP10* gene in women, indicating the possibility that sex-specificity is underlying PTH level. To conclude, findings from this study improve the current knowledge of the genetic factors regulating PTH levels and their validation in independent populations would be beneficial.

## Additional file

Additional file 1: Supporting Information. (XLSX 54 kb)

## Abbreviations

GWAS: genome-wide association study
PTH: Parathyroid hormone
RIA: radio-immunoassay method
SNP: single nucleotide polymorphism
